# Virtual Arthroplasty Follow-Up: Better for the Trust, Patients, and the Planet

**DOI:** 10.7759/cureus.31978

**Published:** 2022-11-28

**Authors:** James D Richards, Michael Stoddart, Benjamin Bolland

**Affiliations:** 1 Trauma and Orthopaedics, Musgrove Park Hospital, Taunton, GBR

**Keywords:** follow-up appointment, net zero, virtual follow-up, hip arthroplasty, arthroplasty, orthopaedics

## Abstract

Background

The Virtual Arthroplasty Follow-Up (VARF) Quality Improvement Project was initiated in March 2020 with the aim of improving patient experience while reducing costs to the trust, the patient, and the planet.

Methodology

This retrospective study was conducted in a district general hospital. Patients were assessed based on their Oxford Hip Score (OHS), University of California, Los Angeles (UCLA) activity score, and an X-ray. A patient satisfaction survey was undertaken via phone call using a 10-point questionnaire. These responses were then correlated to age, distance travelled, and OHS/UCLA scores. The environmental impact was estimated using CO_2_ emissions for driving and outpatient clinics taken from relevant literature.

Results

A total of 132 patients were enrolled in the project. Overall, 75% demonstrated a good outcome from both their X-ray and OHS/UCLA scores. Further, 23% of patients required an additional phone call, of which a further 77% were re-enrolled in the VARF pathway. Only five of 132 (3.8%) patients required a face-to-face review. The patient satisfaction survey enrolled 52 patients, 90% of whom were satisfied with the service. Only one patient stated being dissatisfied with the service. Overall, 77% of patients felt that the service saved them time, money, or both. The strongest predictor of patient satisfaction was the OHS (r = 0.52) where a score of <35 was associated with a nine-fold increase in either responding neutral or dissatisfied with the service. Accounting for both travel and clinic space, approximately 8 tonnes of CO_2_ equivalent were saved. Once time and cost-saving from virtual clinics were included, this project saved the trust £21,408 and patients £948.

Conclusions

VARF has been shown to be an appropriate way to follow up arthroplasty patients which maintains high patient satisfaction while reducing the environmental impact, saving patients’ time and money, and freeing up clinic space for other uses. A potential improvement of the process would be to triage those with low OHS to a more intensive follow-up.

## Introduction

With improving life expectancy and an ageing population, the demand for hip arthroplasty is continuing to rise [1]. This had created sizable demands on outpatient services. It has been acknowledged that, from a clinical perspective, these appointments do not need to be face-to-face [1,2]. Other studies have shown virtual follow-up to be cost-effective for organisations and satisfactory for arthroplasty patients [3,4]. Studies specifically examining the follow-up of fracture clinic patients have also shown considerable financial benefit [5]. Virtual clinic appointments align with the current NHS strategy targeting ‘net zero’ services [6]. To our knowledge, no other paper has shown the benefit to the planet of virtual arthroplasty appointments or correlated data about patient satisfaction.

This project shifted part of the arthroplasty follow-up service to a virtual system called Virtual Arthroplasty Follow-Up (VARF). In this paper, we aimed to examine the outcomes of the service as well as the benefits for the trust, the patients, and the planet. This system aimed to maintain or improve patient experience, reduce face-to-face attendance, improve cost efficiency, and reduce the cost to the environment without any adverse impact on the quality of care.

## Materials and methods

This retrospective cohort analysis was conducted at Musgrove Park Hospital (MPH), Taunton. MPH is a relatively large District General Hospital (DGH) and trauma unit. According to the National Joint Registry (NJR), primary hip arthroplasty makes up our largest group of arthroplasty patients, accounting for 54% of the total [7]. This equated to 144 patients per year from April 2020 to March 2021. However, this year will likely be far lower than normal levels of arthroplasty due to the coronavirus disease 2019 (COVID-19) pandemic.

**Figure 1:**
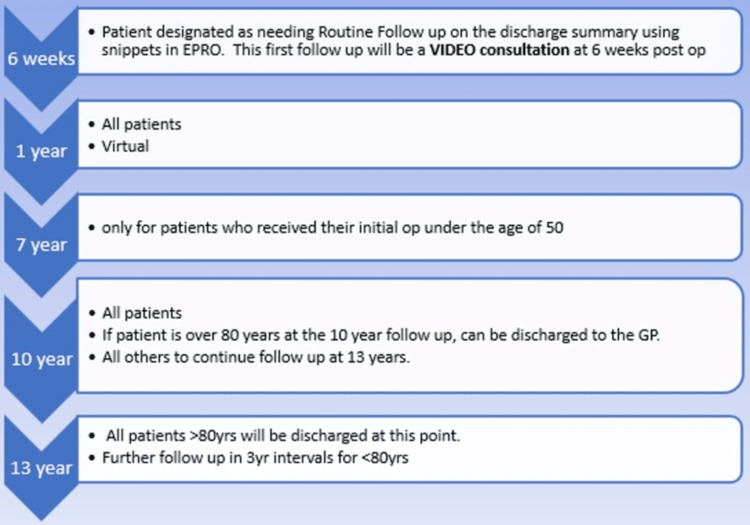
The virtual arthroplasty follow-up pathway. EPRO = a patient record system; Snippets = a standardised template used for arthroplasty patients; yr = year

Our standard hip arthroplasty follow-up process included face-to-face clinic review with a consultant at six weeks, one year, five years, seven years (for patients under the age of 50), 10 years (all patients), and then at three-year intervals for those under the age of 80. This project changed that process to the one shown in Figure 1. The key changes were a virtual follow-up (via telephone) at one year and the removal of the five-year follow-up for all patients to a seven-year follow-up for patients who had their index operation performed at age <50 years.

**Figure 2:**
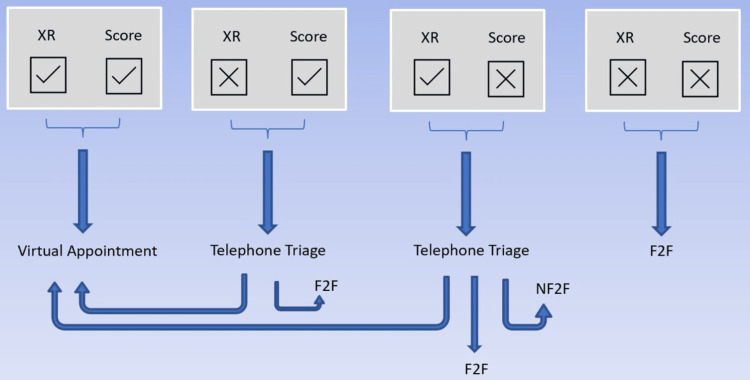
Possible patient pathways at the one-year follow-up point. XR = X-ray; F2F = face-to-face; NF2F = non-face-to-face

Patients were triaged through to virtual follow-up by consultants at their six-week postoperative appointment. All triaged patients approaching their one-year follow-up were sent instructions on how to complete patient-reported outcome measures (PROMs), how to book an X-ray at a local hospital, and a pre-paid return letter. The PROMs included the Oxford Hip Score (OHS) (Appendix A) and the University of California, Los Angeles (UCLA) activity score (Appendix B). The X-ray information is used by clinicians to assess cup and stem position, lucency, lysis, wear, and position change. Upon return of the forms and X-rays, the patients were booked to a virtual arthroplasty clinic according to the flow chart shown in Figure 2.

If patients had an acceptable X-ray and score from PROMs they were booked directly into a VARF clinic. An acceptable PROM was counted as an OHS >33 [8]. An X-ray was deemed unacceptable for VARF if there were any signs of new lucency, change in position, or lysis following a consultant-level review of imaging. If patients had either an unacceptable OHS or X-ray, they received an additional call from an advanced care practitioner and were either booked to a face-to-face clinic or re-enrolled in VARF.

Of the 132 patients who were followed up in VARF, 52 were surveyed via telephone to assess the patient experience. A patient feedback questionnaire (Appendix C) containing a mixture of Likert, multiple-choice, and open questions was utilised. The patient questionnaire assessing patient experience was chosen from a peer-reviewed paper [3]. Additional demographic data for age and distance travelled to the hospital for these patients were collected using information from our electronic patient records system.

Costings for face-to-face and virtual clinic appointments were provided by the orthopaedic management team. They included the cost for use of space, health professional time, and overhead costs.

Local institutional review board approval was obtained for the collection, processing, and publication of this service evaluation project. All data were stored on a secure database for patient confidentiality.

## Results

The breakdown of patient selection is shown in Table 1. At the time of data collection, 240 patients had received a primary total hip replacement (THR) and had been initially enrolled in VARF. At their six-week postoperative review, 52 patients had ongoing face-to-face follow-ups. This was primarily due to problems with other joints or known complications following surgery. In total, 188 patients were put through for VARF assessments at their one-year follow-up point. Of the remaining, there were 34 patients still to be reviewed, and 22 who did not attend (DNA)/moved out of the area or passed away. This left 132 patients who had completed a VARF assessment and were included in data collection.

**Table 1:** Breakdown of patient selection. DNA = did not attend; OOA = out of area

Exclusion criteria	n
Total hip arthroplasty patients	240
On-going face-to-face review	52
Still to be reviewed in the clinic	34
DNA/moved OOA/passed away	22
Total for data collection	132

**Figure 3:**
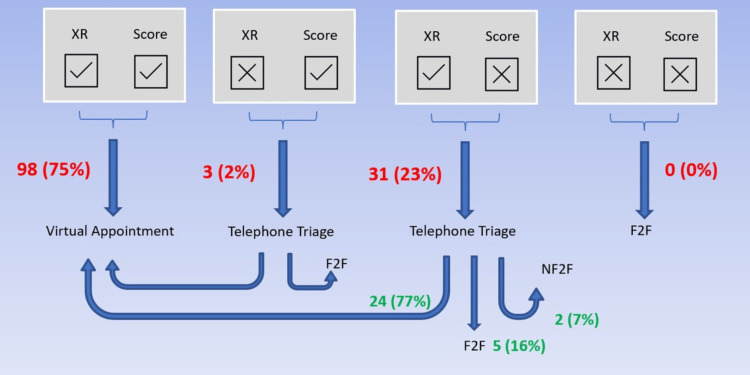
Number of patients in each domain at the one-year follow-up point. XR = X-ray; F2F = face-to-face; Nf2f = non-face-to-face

Of these 132 patients, 98 (75%) had acceptable scores and X-rays and were put directly through to VARF, as shown in Figure 3. In total, 32 (23%) patients had an acceptable X-ray but a low OHS. These patients had an additional phone call from a clinician (Figure 3). In total, 24 of these were then re-introduced to the VARF process. Only five (3.7% of the total) patients received an additional face-to-face appointment. Only three patients had an acceptable score with an ‘unacceptable’ X-ray, who underwent repeat X-rays and scores before their one-year follow-up. No patients had both an unacceptable X-ray and OHS.

Oxford Hip Scores

**Figure 4:**
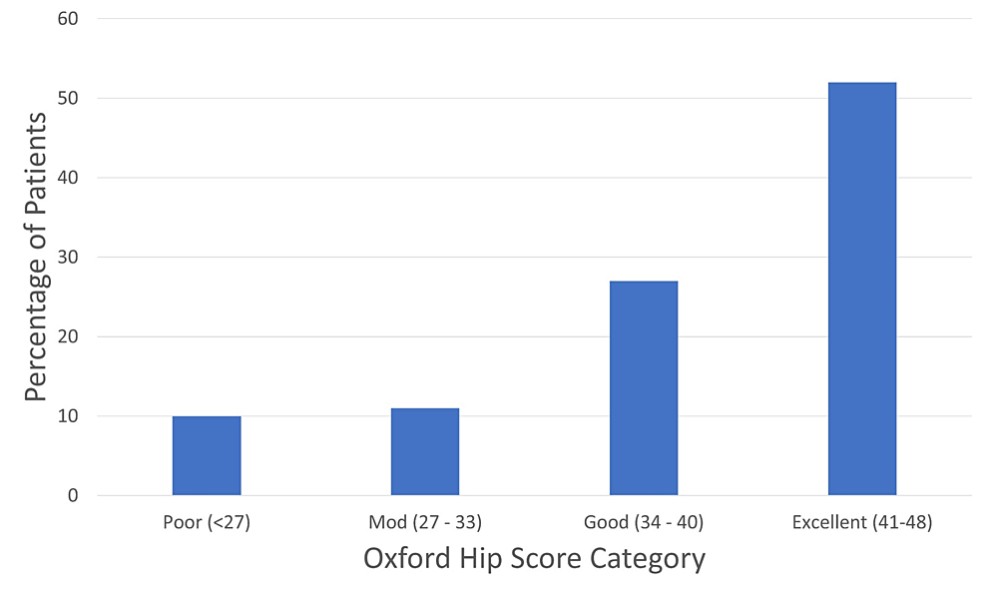
Oxford Hip Scores by outcome category. Mod = moderate

The results from the OHS of patients in VARF are shown in Figure 4. The majority (52%) had an excellent outcome. Only 10% had a poor outcome characterised by a score of less than 27.

UCLA activity score

**Figure 5:**
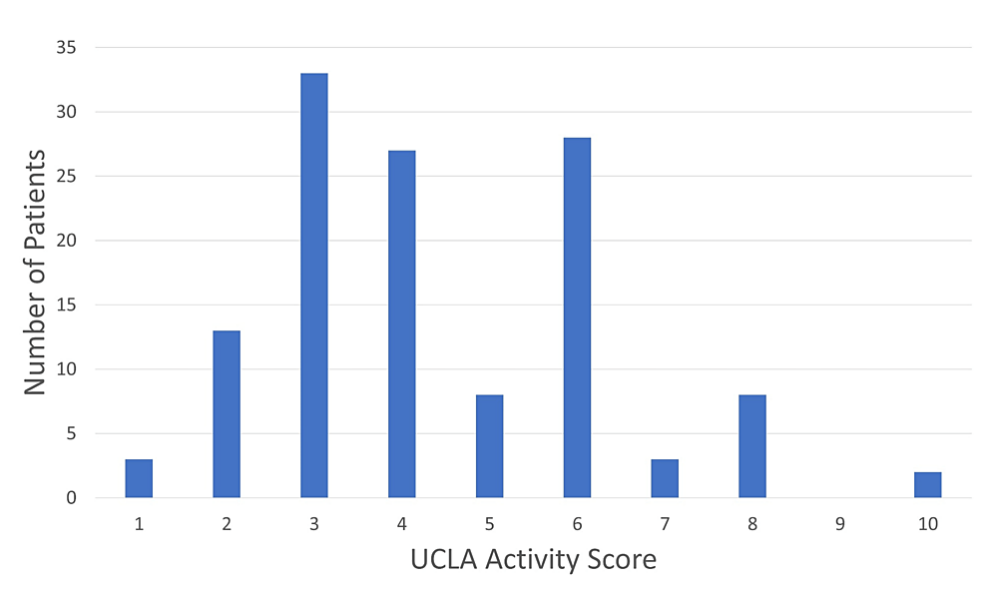
Bar chart showing the number of patients in each domain of the UCLA activity score. UCLA = University of California, Los Angeles

The results of the UCLA activity scores at the VARF clinic are shown in Figure 5. Most patients fell into the bracket between 3 and 6. This describes a range of activities from ‘activities limited to housework and shopping’ to ‘regularly participates in moderate activities including swimming’.

Patient survey data

The patient satisfaction survey (Appendix C) was used to collect data from a total of 52 patients. The mean background data is shown in Table 2.

**Table 2:** Table showing background data for the patient survey group. UCLA = University of California, Los Angeles

Background data	N
Patients contacted	52
Mean age (years)	75.5
Mean distance travelled (miles)	24.6
Mean Oxford Hip Score (/48)	36.9
Mean UCLA activity score (/10)	4.19

**Figure 6:**
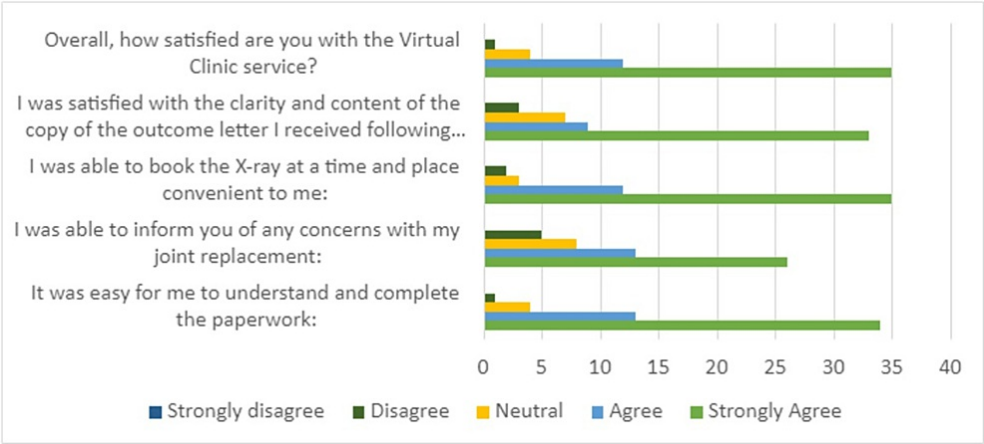
Response rates to five Likert questions.

The data for the Likert questions are shown in Figure 6. The key findings from these questions were that 90% of patients either agreed or strongly agreed with the question ‘Overall, how satisfied are you with the virtual clinic service?’. Overall, 67% strongly agreed with this statement. Only one patient stated that they disagreed with this statement.

The data for the question ‘Do you feel the virtual clinic saved you any time or money compared to a standard outpatient appointment?’ are shown in Table 3. Overall, 77% of patients stated that the virtual clinic had at least some benefit.

**Table 3:** Response rates for question ‘Do you feel the virtual clinic saved you any time or money compared to a standard outpatient appointment?’.

Do you feel the virtual clinic saved you any time or money compared to a standard outpatient appointment?	
Both	25 (48%)
Time	14 (27%)
Money	1 (2%)
Neither	12 (23%)

The answers to question 9, namely, follow-up preferences, are shown in Table 4. Interestingly, one-third of patients were happy to be followed up by their general practitioner (GP) and referred to secondary care as needed. The other key finding was that almost one-third of patients preferred a face-to-face follow-up. Further analysis of this group showed that 11 of the 17 had stated that they were satisfied with the VARF process. All patients who had an overall neutral or dissatisfied opinion of VARF wanted to have face-to-face follow-ups.

**Table 4:** Response rates for the question ‘follow-up preferences’. GP = general practitioner

Follow-up preferences	
Further phone call	37%
With GP	33%
Face-to-face	29%
Video	1%

Correlational data

**Figure 7:**
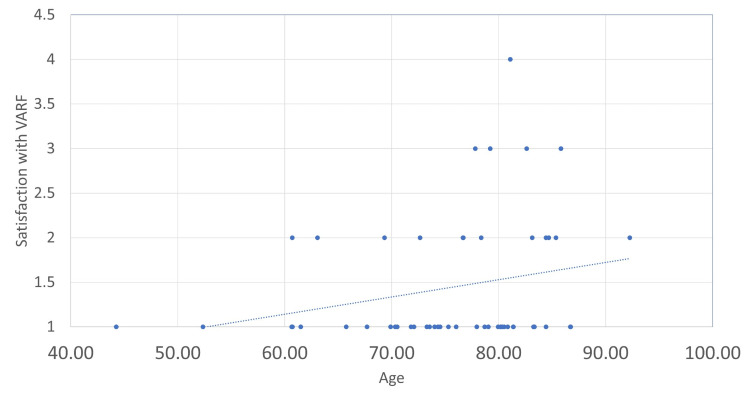
Age versus overall satisfaction with VARF. Weak correlation, r = 0.25. VARF = Virtual Arthroplasty Follow-Up

**Figure 8:**
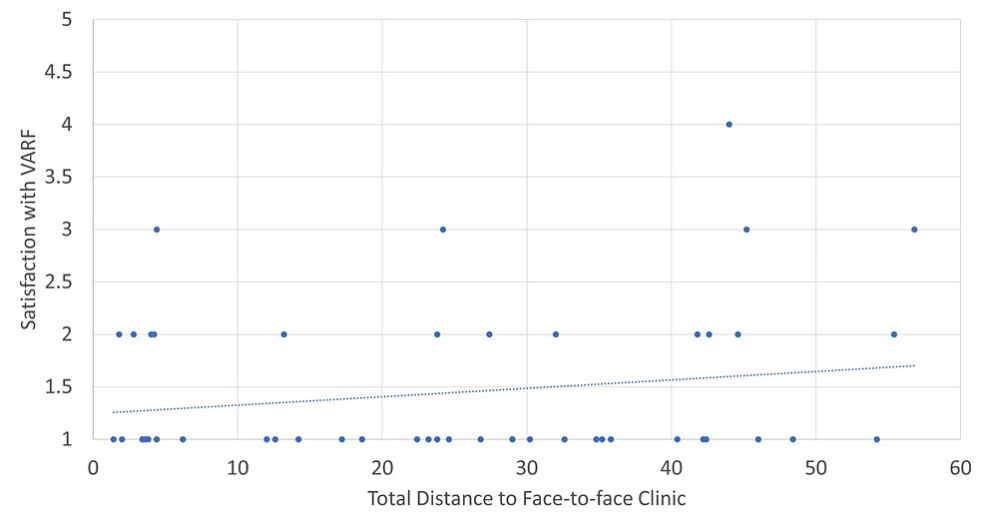
Distance travelled versus satisfaction with VARF. Weak correlation, r = 0.19. VARF = Virtual Arthroplasty Follow-Up

**Figure 9:**
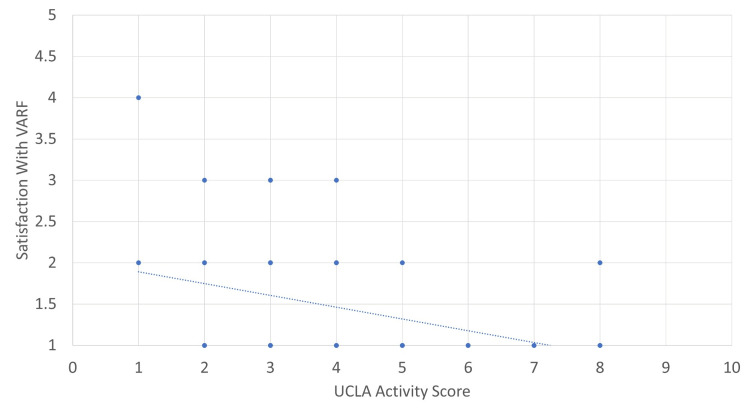
UCLA activity score versus overall satisfaction. Moderate correlation, r = 0.38. VARF = Virtual Arthroplasty Follow-Up; UCLA = University of California, Los Angeles

**Figure 10:**
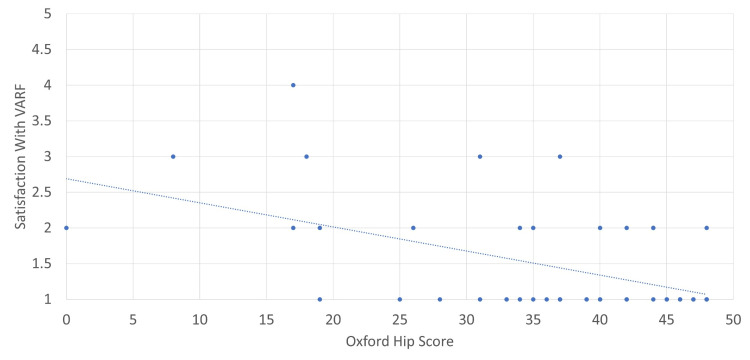
Oxford Hip Score versus overall satisfaction with VARF. Strong correlation, r = 0.52. VARF = Virtual Arthroplasty Follow-Up

Using the additional demographic data gained from those who participated in the patient survey, we could correlate satisfaction with the VARF service with different measures. In Figures 7-10, a score of 1 reflects ‘very satisfied’ and a score of 5 reflects ‘very dissatisfied’ with the service. Correlations were calculated using Pearson’s correlation coefficient where an r of 1 is a perfect correlation between two variables. Correlations were relatively weak for both distance travelled and age, with increasing age and distance travelled offering very mild predictive value for satisfaction. Both the PROMs used provided a much stronger correlation. The OHS had an r of 0.52. Further analysis of this data showed that patients with a score of below 35 were nine times more likely to be either neutral or unsatisfied with the service.

Environmental benefits

The 52 patients surveyed would have travelled an average of 24.6 miles total (to and from the hospital) for their clinic appointments. This totals 1,279 miles not driven. If all drive the equivalent of an average petrol car, this equates to 358 kg of CO_2_e (carbon dioxide equivalent) saved [9]. Additionally, the use of face-to-face clinic space comes with an associated environmental cost for the lighting, heating, and waste generated. This has previously been calculated to be between 56 and 76 kg of CO_2_e per clinic slot [5,10]. Totalling between 2,912 and 3,952 kg of CO_2_e for this group. This brings the total carbon saving for just these 52 patients to a lower estimate of 3,270 kg of CO_2_e or 62.9 kg CO_2_e per patient per appointment. If we upscale these averages to the entire group of 132 VARF patients, this would equate to over 8 tonnes of CO_2_e saved.

Financial benefits to patients

According to the Royal Automobile Club (RAC), the cost per mile to drive a petrol car (50 mpg efficiency) is 10.9p when petrol prices are at £1.20/l [11]. The mean distance travelled (24.6 miles) totalled £2.68 in petrol costs. The parking cost at MPH for two hours is £4.50. This saves approximately a total of £7.18 per patient per appointment and a total of £948 for all VARF patients. We accept that this is an estimate and assume that all patients drive to their appointments. Previous studies at this hospital have shown that approximately 82% of patients drive to their clinic appointments [5].

Financial benefits to the trust

There is a sizeable difference between the cost of a face-to-face (£1,922) and non-face-to-face (£446) appointment for the trust, totalling £1,476 per clinic list. These numbers include staff time, overheads, and use of the space. Additionally, VARF appointments are scheduled as 10 minutes shorter than their face-to-face counterparts (scheduled as 20 minutes). The standard clinic session is 210 minutes. For a full face-to-face clinic service for 132 patients, this would require 12.6 consultant clinics at a cost of £24,217. If all patients had a VARF follow-up, this would require 6.3 consultant clinics at a cost of £2,809, a £21,408 saving. Once time and space are accounted for this represents almost a 90% saving for the department using VARF rather than full face-to-face outpatient appointments. We acknowledge that this is not necessarily a true saving for the trust as the space will still be heated, lit, and staffed. It does allow for better use of resources for clinics that require face-to-face review.

## Discussion

Our study has shown that the VARF process is clinically viable for following up patients. Overall, 75% of patients were put directly through to virtual follow-up. Of the remaining patients, less than 4% required a face-to-face follow-up following triage by a healthcare professional. Moreover, 79% of the total patients had good or excellent outcomes based on their OHS. One of the key clinical concerns for following up patients non-face-to-face is that those who may need early revision are missed. At this point of follow-up, our study did not highlight any missed patients. This data is supported by other studies [3,4,12,13]. A large study on patients who received a hemi-arthroplasty showed that it is safe for patients to self-report issues without formal follow-up [14].

In our study, we found that almost 90% of patients stated that they were either satisfied or very satisfied with the service. This is comparable with other studies of virtual follow-up [3]. Some other studies have shown that while satisfaction with virtual follow-up is high it is exceeded by face-to-face follow-up [12]. This data is perhaps supported by our patients’ preference for future follow-up, with only 37% stating they would want to continue phone call follow-up. While this group does outsize those who would prefer ongoing face-to-face follow-up (33%), this is not an insignificant number. Further analysis of this group showed that approximately two-thirds of this subgroup were satisfied with their follow-up, indicating that the preference for a face-to-face review is relatively weak. Interestingly, one-third of the patients surveyed indicated that they would happily be followed up by their GP rather than the hospital. The reasons for this are unclear. One potential reason is the ease of access and familiarity with their own GP.

Response rates are another concern. This study did not include this data. Other studies have shown response rates varying from 76% to 92% [3,4,12]. This higher response rate is comparable with that of face-to-face appointments who have a DNA rate of approximately 8% [15]. Some other studies have shown that virtual follow-up is associated with a reduction in DNAs [16,17]. One study showed that 24% of eligible patients cited lack of internet access as their reason for declining virtual follow-up [12]. The mean age of declining follow-up, for this reason, was 74 years [12]. Given that we do not routinely follow up those aged 80 and over and there is increasing technological literacy among older generations, we believe this will not be an ongoing issue.

There are sizeable benefits for the trust, the patients, and the planet from a virtual arthroplasty service. This study made a potential cost saving for the trust of approximately £21,408 (£162.2 per patient enrolled), as well as over 8 tonnes of CO_2_e equivalent (62.9 kg CO2e per patient enrolled) just for the patients included. Similar cost savings have been found in other studies [3,4,12]. Additionally, it saved the patients involved almost £1,000 not including the time taken to attend an appointment. This time saving is difficult to account for but is not insignificant with 75% of our patients stating time as at least one of the benefits of a virtual service. Comparatively, only 2% stated that money alone was the benefit. The time-saving benefit has been reflected in a wider NHS study with time off work and childcare cited as key issues [17].

Limitations of our study are that it is a retrospective cohort analysis and we have not directly compared a similar patient group who underwent a standard face-to-face follow-up. Because our process for obtaining satisfaction surveys was not fully randomised, this may not account for a representative patient group. Lastly, the patient survey was not validated but was created by several arthroplasty consultants and clinical nurse practitioners. To our knowledge, there is no formally validated survey available for virtual arthroplasty patients. Our survey is comparable with those seen in other surveys [3].

## Conclusions

Our study has shown that VARF is a clinically safe process that maintains patient satisfaction and has significant benefits for the trust as well as for the planet. While more research is needed, we believe that with careful patient selection, virtual follow-up is the most sustainable model of care.

## Figures and Tables

**Figure 11:**
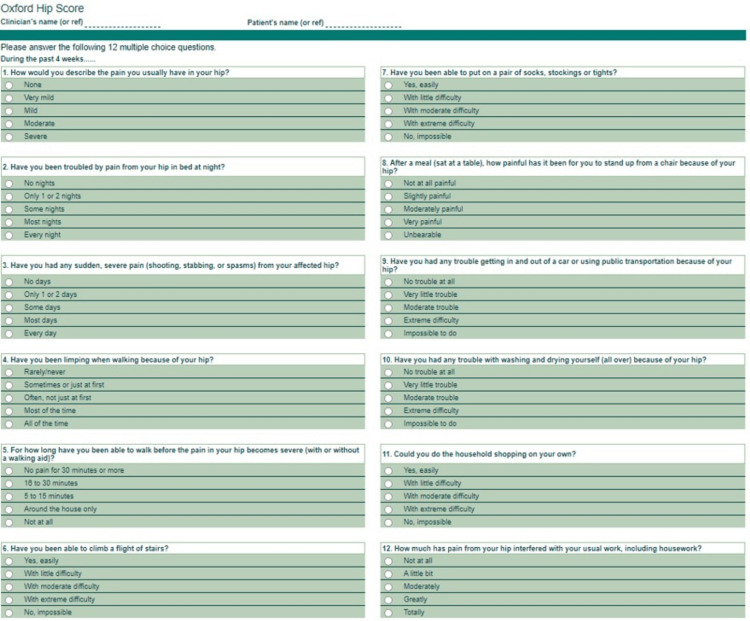
Oxford Hip Score.

**Figure 12:**
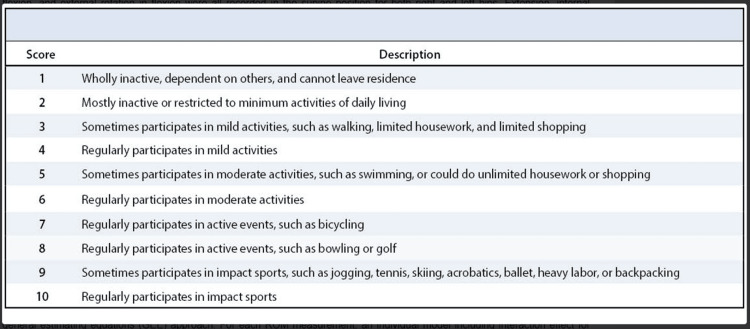
University of California, Los Angeles activity score.

## Appendices

Appendix A

Appendix B

Appendix C

Patient Satisfaction Questionnaire (VARF patients January-December 2021)

(1) It was easy for me to understand and complete the paperwork:

Strongly agree      Agree      Neither agree nor disagree      Disagree Strongly      Disagree

(2) I was able to inform you of any concerns with my joint replacement(s) (this may also include via a requested telephone call):

Strongly agree      Agree      Neither agree nor disagree      Disagree Strongly      Disagree

(3) I was able to book the X-ray at a time and place convenient to me:

Strongly agree      Agree      Neither agree nor disagree      Disagree Strongly      Disagree

(4) I was satisfied with the clarity and content of the copy of the outcome letter I received following Virtual Clinic review:

Strongly agree      Agree      Neither agree nor disagree      Disagree Strongly      Disagree

(5) Did you also receive a telephone call to further discuss the joint replacement?

Yes, please continue below; No, please move to Q6

I was satisfied with the outcome of the telephone call:

Strongly agree      Agree      Neither agree nor disagree      Disagree Strongly      Disagree

(6) Did you also attend an outpatient appointment following on from, or instead of, Virtual Clinic review?

Yes, please continue below; No, please move to Q7

I was satisfied with the outcome at the Orthopedic Outpatient Appointment following further assessment of the joint replacements(s):

Strongly agree      Agree      Neither agree nor disagree      Disagree Strongly      Disagree

The appointment was with:

An orthopedic surgeon      A specialist physiotherapist

(7) Do you Feel the Virtual Clinic saved you any time or money compared to a standard outpatient appointment?

Time      Money      Both      Neither

If yes, please tick all reasons that apply:

Travel distance      Travel time      Travel costs      Parking costs      Time off work Less wait time in clinic

Other (please give details)…………………………………………………

(8) Overall, how satisfied are you with the Virtual Clinic service?

Very satisfied      Satisfied      Neither satisfied nor dissatisfied      Dissatisfied      Very dissatisfied

(9) If you have any comments on how we could improve the service, please tell us (please continue on the back page if you like)

10) What would be your preferred method of future routine joint replacement review (normally 1, 7, and 10 years following surgery, then every 3 years)

- Virtual Clinic (as the previous review, with option of formal appointment if concerns)

- Web-based follow-up (email and electronic questionnaires to complete on a computer, tablet, or suitable phone, with the option of formal appointment if concerns)

- Formal outpatient clinic appointment at Musgrove Park Hospital

- None, I would rather be discharged to the care of my GP
